# Sub-monolayer film growth of a volatile lanthanide complex on metallic surfaces

**DOI:** 10.3762/bjnano.6.248

**Published:** 2015-12-16

**Authors:** Hironari Isshiki, Jinjie Chen, Kevin Edelmann, Wulf Wulfhekel

**Affiliations:** 1 Physikalisches Institut, Karlsruhe Institute of Technology (KIT), Wolfgang-Gaede-Straße 1, 76131 Karlsruhe, Germany,; 2 Institut for Nanotechnology, Karlsruhe Institute of Technology (KIT), Hermann-von-Helmholtz-Platz 1, 76344 Karlsruhe, Germany

**Keywords:** β-diketonate, molecular films, scanning tunneling microscopy, terbium, volatile lanthanide complex

## Abstract

We deposited a volatile lanthanide complex, tris(2,2,6,6-tetramethyl-3,5-heptanedionato)terbium(III), onto metal surfaces of Cu(111), Ag(111) and Au(111) in vacuum and observed well-ordered sub-monolayer films with low temperature (5 K) scanning tunneling microscopy. The films show a distorted three-fold symmetry with a commensurate structure. Scanning tunneling spectroscopy reveals molecular orbitals delocalized on the ligands of the molecule. Our results imply that this complex can be transferred onto the metal substrates without molecular decomposition or contamination of the surface. This new rare-earth-based class of molecules broadens the choice of molecular magnets to study with scanning tunneling microscopy.

## Introduction

Several carefully designed metal-organic molecules with magnetic-ion cores exhibit long spin-relaxation times for the reversal of the localized magnetic moment at low temperatures. These molecules are called single-molecule magnets (SMMs), because the magnetic properties are attributed to the individual single molecules as a result of the quantum mechanical interaction of the local magnetic moment and the ligand field [1]. Lanthanide-based SMMs have attracted much interest due to the potentially huge energy barrier for the reversal of magnetization [2]. This is an advantage from an applications viewpoint for magnetic storage devices based on single molecules. The investigation of individual molecules is crucial to understand the physics of SMMs. Scanning tunneling microscopy (STM) is one of few methods that can reveal the magnetic properties on the level of single molecules. The magnetic properties of single ions and single molecules of transition metals have been widely investigated with STM [3–6]. However, there are few studies on lanthanide-based SMMs with low-temperature STM [7–8] despite the variety of reports of their peculiar magnetic properties. One plausible reason is the difficulty in transferring them onto substrates in an ordered and clean way. Because of the huge molecular mass of lanthanide-based SMMs, many of them cannot be sublimed without decomposition, even in vacuum. Some studies have suggested methods other than vacuum deposition to solve the problem, but they are limited by the resulting level of contamination of the surface [9]. Finding a class of rare-earth SMMs that can be suitably transferred would be a significant step forward towards the application of magnetic molecular devices in which well-defined homogeneous molecular structures are required.

**Figure 1:**
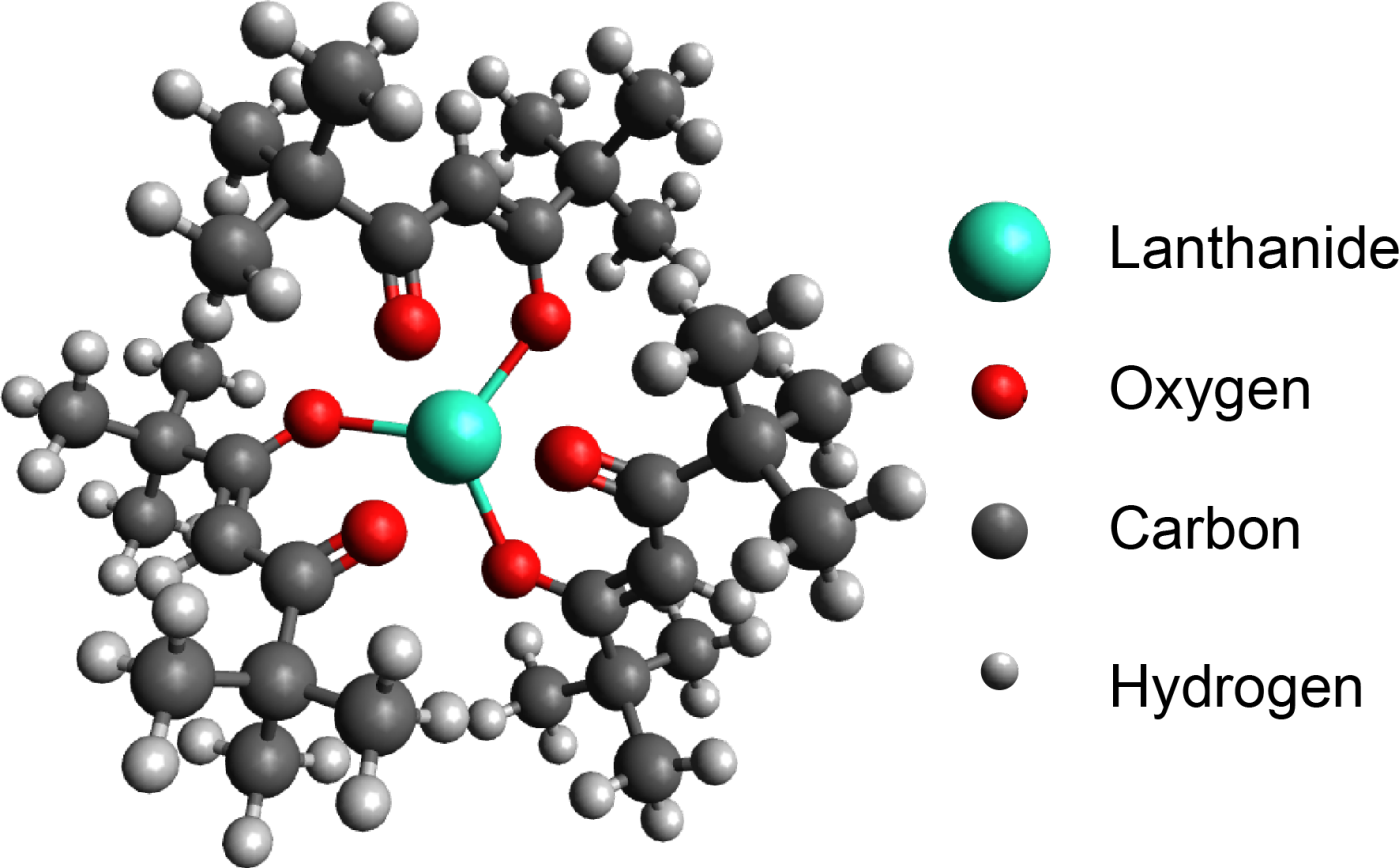
A ball–stick model of tris(2,2,6,6-tetramethyl-3,5-heptanedionato) lanthanide(III).

The β-dikenonate lanthanide(III) series have been known as volatile lanthanide complexes since the 1970s and have been used as precursors in chemical vapor deposition in the thin-film industry [10]. However, to the best of our knowledge, there has been only one paper that reports on the characterization of isolated molecules of ruthenium (platinum group metal) β-dikenonate with low-temperature STM [11]. Tris(2,2,6,6-tetramethyl-3,5-heptanedionato)lanthanide(III) (Ln(thd)_3_) has the simplest molecular structure among them and is highly volatile thermally stability [12–13]. In this molecule, the lanthanide(III) ion is coordinated by three β-dikenonate ligands (see Figure 1) and the total charge of the molecule is zero. Recent DFT calculations for Ln(thd)_3_ in gas phase show that the D_3_ symmetry structure corresponds to the minimum of the potential energy [14]. Though remarkable magnetic properties of Ln(thd)_3_ in bulk have not been reported, some of their derivatives exhibit SMM behavior [15]. It thus might be interesting to investigate Ln(thd)_3_ with low-temperature STM. In this work, we chose Tb(thd)_3_ (Tb = terbium) and deposited molecules on Au(111), Ag(111) and Cu(111) surfaces to explore the transferability of lanthanide molecules onto metal surfaces without decomposition.

## Results and Discussion

**Figure 2:**
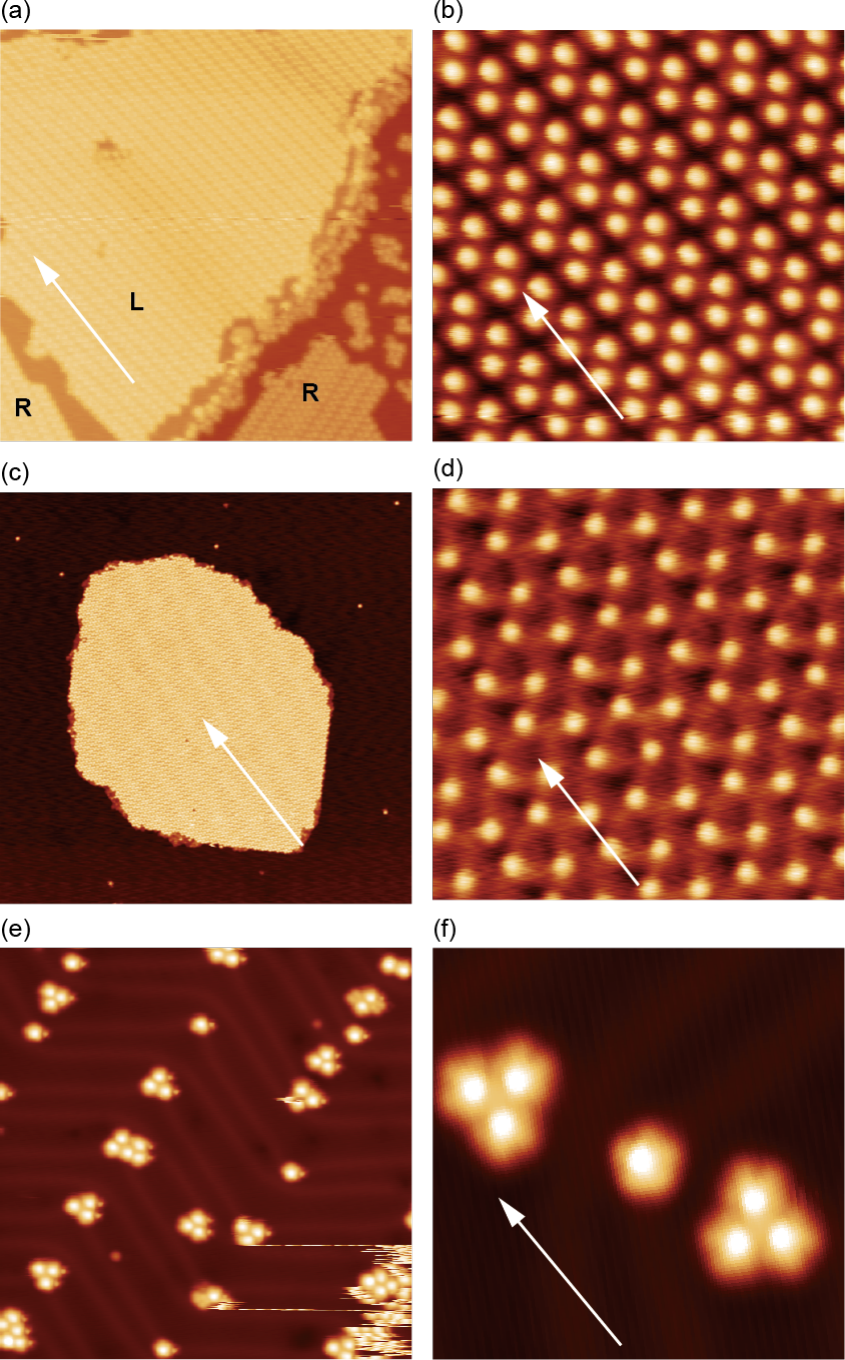
STM topographies of Tb(thd)_3_ (a,b) on Cu(111), (c,d) on Ag(111), and (e,f) on Au(111). The image sizes are (a) 50 × 50 nm^2^, (c) 180 × 180 nm^2^, (e) 40 × 40 nm^2^ and (b,d,f) 10 × 10 nm^2^. The white arrows indicate the 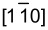 direction of the (111) surfaces. The labels R and L in (a) show the chirality of the film. Set point: (a,b) −0.8 V, 70 pA, (c) −0.8 V, 100 pA, (d) −1 V, 50 pA, (e,f) 1 V, 50 pA.

Figure 2a–f shows STM topographies of Tb(thd)_3_ deposited on Cu(111), Ag(111) and Au(111), respectively. The imaging parameters are given in the caption. The white arrows indicate the 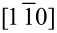 direction, which was determined from images with atomic resolution of each surface. We observed large molecular islands of Tb(thd)_3_ growing homogeneously on Cu(111) and Ag(111) and very few isolated molecules on these substrates (see Figure 2a,c). Thus, the deposited molecules diffuse at room temperature on the substrate, and eventually, nucleation of islands occurs due to an attractive molecule–molecule interaction, resulting in sub-monolayer film growth. The island height relative to the substrate in the topography is ≈340 pm at 1 V sample bias, but depends on the sample bias in a sensitive manner. Additionally, on Au(111), we observed isolated molecules and small clusters at the elbow sites of the Au(111) herringbone reconstruction [16], but no large islands at this coverage (see Figure 2e). Figure 2f shows an isolated molecule in the center, and two clusters containing three molecules (i.e., molecular trimers). The cluster formation at the elbow sites of the herringbone indicates higher adsorption energy at these sites. The molecules can attach here but cannot diffuse further on the surface to form larger islands at room temperature. This preferential nucleation at the elbow sites has been reported for other molecules [17–18].

**Figure 3:**
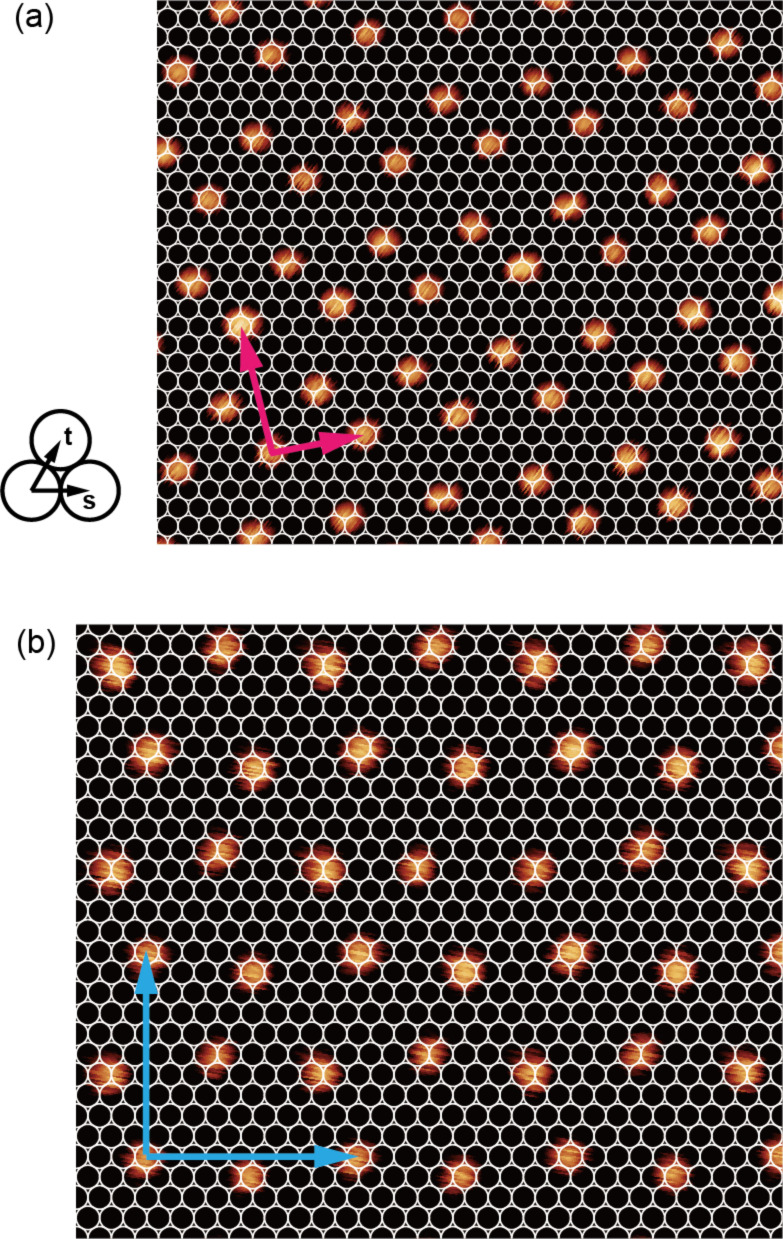
STM topographies of Tb(thd)_3_ films (a) on Cu(111) and (b) on Ag(111) with the model of the substrate lattices. The white circles represent Cu or Ag atoms. The nearest neighbor distance of Cu (Ag) atoms of substrate is 255 pm (288 pm) in this model. The vector **t** and **s** shown in left lower of (a) are lattice vector of the substrate. The unit vector of films are shown as red and blue allows in each panel.

In the extended islands on Cu(111) and Ag(111), a quasi-triangular lattice composed of molecules is observed (see Figure 2b,d). On Cu(111), we observed a particular row-like structure: two-molecule-wide rows are formed separated by dark stripes along a direction which is +11° rotated from 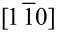 (see Figure 2b). Looking at Figure 2a closely, we find two kinds of films, labeled by R and L, which are −11° and +11° rotated from the 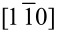 direction, respectively. The domains are related by a mirror operation along 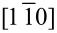. Thus, taking into account the symmetry of the substrate, the two domains represent opposite chirality regarding the full structure of substrate and film. To investigate the superstructure of the molecular films, we superimposed a schematic model of Cu(111) and Ag(111) lattices on STM topographies of the films with enhanced contrast, as shown in Figure 3a,b. The analysis reveals that the molecular films assemble in a commensurate manner. Note that the adsorption site of the molecules with respect to the lateral position (on top, three-fold hollow site, etc.) could not be determined in our model since we were unable to obtain atomically resolved images of the substrates and molecules at the same time. The unit cells can nevertheless be represented by using vector **s** and **t** (**s’** and **t’**) for the lattice vector of Cu(111) (Ag(111)) as follows:

[1]
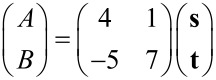


for films on Cu(111) and,

[2]
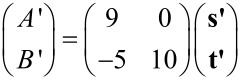


for films on Ag(111). The complicated structure of the molecular films is the result of the competition between intermolecular interactions and molecule–substrate interactions. The creation of these commensurate lattices implies that the molecules are transferred onto substrates without decomposition and a relatively strong molecule–substrate interaction is maintained.

**Figure 4:**
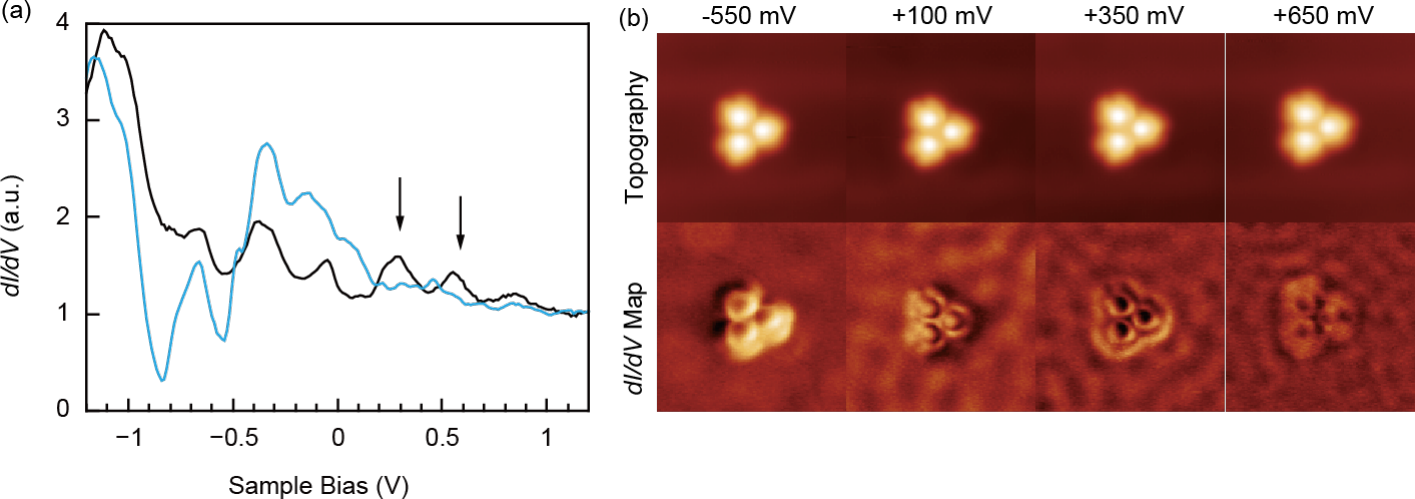
(a) *dI/dV* spectra of a molecule in a trimer (black line) and on the Au substrate (blue line). (b) STM topography and *dI/dV* maps at the indicated voltages.

We took *dI/dV* spectra on a molecule in a trimer on Au(111) to determine the local density of state (DOS) of the adsorbed molecules. The spectra of a molecule (black line) and the Au substrate (blue line) are shown in Figure 4a. The most prominent features in the spectra are marked by arrows at +350 mV and +650 mV. The peak at about −400 mV on Au(111) is caused by the surface state of gold [19]. To see the particular distribution of the DOS of the molecules, we performed *dI/dV* mapping on the trimer. The *dI/dV* maps at bias voltages of −550 mV, +100 mV, +350 mV and +650 mV, together with their corresponding topographies, are shown in Figure 4b. The corrugation of the DOS on the substrate represents the standing waves caused by impurity scattering of the electrons of the surface state [20]. At +350 mV and +650 mV, where the maxima in the *dI/dV* spectrum were found, the DOS is localized on the molecules, that is, the shape represents the molecular orbitals. These significantly differ from the topography (e.g., the center of the molecules is bright in the topography, but it is dark in the *dI/dV* map). At the voltages other than +350 mV and +650 mV, the shapes of the local DOS are more similar to the topographic images. This implies that the topographic images more likely represent the geometrical height of the molecules.

## Conclusion

We showed that Tb(thd)_3_ complexes can be transferred onto metallic substrates by sublimation without decomposition or surface contamination. Furthermore, we showed that well-ordered monolayer films of lanthanide molecules are formed on Cu(111) and Ag(111). Due to the chemical similarity of all rare-earth elements, we speculate that similar complexes with different lanthanide cores can be sublimed as well. Thus, with this work, we have broadened the molecular species available for the study of rare-earth molecular magnetism.

## Experimental

All experiments other than the molecule deposition and Ar sputtering were done in ultra-high vacuum conditions (≈10^−10^ mbar). The Tb(thd)_3_ compound of 99% purity was purchased from Alfa Aesar and degassing was carefully performed by heating to ≈325 K in a ceramic crucible for hours prior to evaporation. The Cu(111), Ag(111) and Au(111) single crystal substrates were cleaned with a standard Ar sputtering and annealing processes in a separate preparation chamber. After annealing and cooling down to room temperature, the substrates were transferred to a molecule deposition chamber and were exposed to a molecule flow of ≈0.1 monolayers/s for several seconds at a sublimation temperature of ≈335 K. The pressure during deposition was ≈8 × 10^−8^ mbar. After deposition, the samples were immediately transferred to the STM chamber and cooled down to 5 K. The measurements were done with a homebuilt STM [21]. During the measurement, the sample temperature was kept at ≈5 K. The *dI/dV* spectra were taken using a standard lock-in amplifier technique with a 487 Hz modulation frequency and 20 mV modulation voltage with an open feedback loop. The *dI/dV* maps were recorded with the same lock-in parameters but with a closed feedback loop.
